# Ensemble deep learning approach for traffic video analytics in edge computing

**DOI:** 10.1038/s41598-025-25628-7

**Published:** 2026-01-03

**Authors:** Malathy Sathyamoorthy, Vani Rajasekar, Sathya Krishnamoorthi, Dragan Pamucar

**Affiliations:** 1 Department of Information Technology, KPR Institute of Engineering and Technology, Coimbatore, India; 2 School of Computer Science and Engineering, Vellore Institute of Technology, Vellore, India; 3 Department of Computer Science and Engineering, Kongu Engineering College, Erode, India; 4 Széchenyi István University, Győr, Hungary

**Keywords:** Tiny YOLO, YOLOR, F-RNN, Video analytics, Ensemble learning, Deep learning, Engineering, Energy infrastructure

## Abstract

Video analytics is the new era of computer vision in identifying and classifying objects. Traffic surveillance videos can be analysed to using computer vision to comprehend the road traffic. Monitoring the real-time road traffic is essential to control them. Computer vision helps in identifying the vehicles on the road, but the present techniques either perform the video analysis on the cloud platform or the edge platform. The former introduces more delay in processing while controlling is needed in real-time, the latter is not accurate in estimating the current road traffic. YOLO algorithms are the most notable ones for efficient real-time object detection. To make such object detections feasible in lightweight environments, its tinier version called Tiny YOLO is used. Edge computing is the efficient framework to have its computation done on the edge of the physical layer without the need to move data into the cloud to reduce latency. A novel hybrid model of vehicle detection and classification using Tiny YOLO and YOLOR is constructed at the edge layer. This hybrid model processes the video frames at a higher rate and produces the traffic estimate. The numerical traffic volume is sent to Ensemble Learning in Traffic Video Analytics (ELITVA) which uses F-RNN to make decisions in reducing the traffic flow seamlessly. The experimental results performed on drone dataset captured at road signals show an increase in precision by 13.8%, accuracy by 4.8%, recall by 17.4%, F1 score by 19.9%, and frame rate processing by 12.8% compared to other existing traffic surveillance systems and efficient controlling of road traffic.

## Introduction

 Surveillance of traffic flow constitutes a critical task in any kind of area. Traffic monitoring should be responsive and alert to uncertain conditions that prevail on the road. Most of the smart city systems have checkpoints at every possible signal on the road to capture and analyse the dynamics of road traffic. Videos captured on consumer electronics like CCTV need to be analysed to understand the statistics of current traffic flow. This data can be effectively used in making dynamic decisions related to controlling traffic. Many video analytical ideas revolve around multiple use cases. To make them use in real-time traffic monitoring, the analysis needs to be done both accurately and faster. This can be achieved with the use of computer vision and deep learning techniques. Computers acquire knowledge of objects in real-time by analysing their videos with the help of Computer Vision. Computer vision incorporates Artificial Intelligence (AI) to detect real-world objects from images and videos. Many researchers have used various forms of AI algorithms to detect objects and gather information about those objects from images and videos^1–3^. In other forms of deep learning algorithms like CNN^4,5^, and RNN^6^, image object detection has become much easier and found its application in numerous fields. To make computer vision a go-to solution for real-world object detection and tracking problems, researchers started to use deep learning models to process multiple images which are sequences from a video to detect and track objects.

While processing videos for the analysis and detection of objects, the frames are split and not all frames are utilized. This may increase the computational cost and time. To have effective detection, the key is to identify the primary frames that are sufficient enough to identify objects without losing any data^7,8^. To have effective use of object detection from videos in real-time, many object detection algorithms were developed. Existing algorithms make use of machine learning models as their backbone. These models had high latency, less accuracy and poor feature extraction owing to dynamic changes in the frames including occlusion lengths, illumination dynamics and so on. Machine learning models tend to observe the key features and extract them to be compared with the reference frame. These techniques were later not useful in real-time use cases that require faster processing of frames and detecting moving objects.

Deep learning (DL) came to light after some years. Deep learning is one form of machine learning with deeper layers of neural networks. These deeper layers make the model imitate the human brain in complex analysis and make them learn on their own. DL algorithms can perform complex operations like analysing patterns, finding similarities, and improving their learning by observing the previous outputs. DL algorithms make the best choice for object detection in real-time videos. Some of the most notable object detection algorithms are Single Shot Detection (SSD), Region-based CNN (R-CNN), You Only Look Once (YOLO), and Faster R-CNN^9,10^. All these algorithms have their deep convolutional neural network with capabilities to extract information about objects in an image with better accuracy. The general procedure in all DL algorithms is almost similar.

The selected frame which is now equivalent to an image is divided into regions and regions of interest that contain the object are identified. The regions which are suspected to hold the object are sent to the next layers while the remaining regions are ignored. The regions are then evaluated for their confidence score and key features of the object are extracted to classify an object based on the pre-trained model. These algorithms have different characteristics in varying numerical values. Hence one should be aware of choosing which object algorithm is best for their use. In road traffic video surveillance, the processing of videos and analysis are done on the cloud system. The latency introduced in sending the video to the cloud and getting the analysis done is not acceptable. To take advantage of edge computing, smaller processing and computing resources are added at the edge and closely tied to the initial source of video. The computation is done on the edge layer and numerical results are conveyed to the cloud server for making decisions like change of route, informing ahead of heavy traffic, dynamic change of speed limits, and modifying the signal waiting timer to quickly respond to traffic flow. This paper focuses on traffic flow surveillance in terms of detecting vehicles and classification from surveillance videos in edge computing scenarios. The monitoring and controlling of real-time traffic from the surveillance cameras is implemented in two steps. The first step performs vehicle detection and classification along with numerical statistics in the edge layer with sophisticated computing resources. The numerical statistics include the type of vehicle and number of each type on the road. The estimated traffic result is sent to the server maintained in cloud which receives the traffic data and with the ensemble learning, responses are made to control the traffic in specified areas. The object detection algorithm implemented on the edge device must be lightweight and have faster processing capability as the surveillance is made in real-time. One such algorithm is the Tiny-YOLO^11^. This algorithm is most suitable for embedded systems with restricted resources^12,13^. To improve the vehicle classification results, YOLO-R^14,15^ is combined with Tiny-YOLO.

**Fig. 1:**
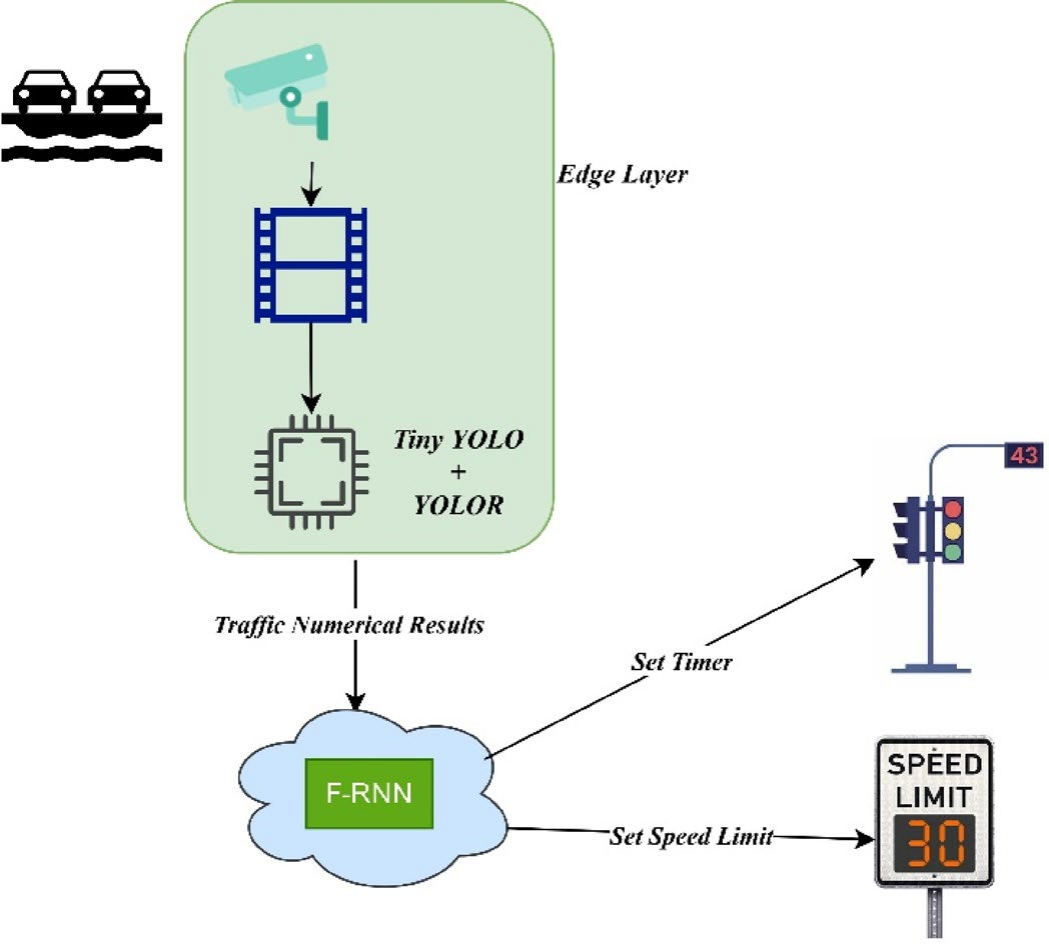
Block diagram of Proposed ELITVA.

This proposed framework of Ensemble Learning in Traffic Video Analytics (ELITVA) is illustrated in Figure. 1. The edge layer is responsible for the detection of vehicles and classifying them into class categories. The cloud layer ensembles the acquired knowledge with the inherent knowledge of Fast-Recurrent Neural Network (F-RNN) to make decisions based on the numerical data.

The significant objectives made in this manuscript are:

- Data pre-processing and selection of efficient features in the drone dataset.
- Implementation of ELITVA for object detection using Tiny YOLO in Edge layer.
- Implementation of YOLOR with explicit and implicit knowledge for object detection.
- Construct an ensemble model of F-RNN on the cloud for traffic video analysis.
- Deter road traffic with the decisions made.

The remainder of this paper will have a literature study in Sect. 2 and Sect. 3 includes the methods and materials. Section 4 explains the working of the proposed ensemble learning architecture. Section 5 analyses the experimental results of proposed ensemble learning and conclusions are stated in Sect. 6.

### Literature study

This section discusses the related works researched so far in the domain of object detection, traffic surveillance and their use in edge computing environments.

### Object detection

Initial object detection algorithms were used only to detect objects in static images. Static image is divided into local parts and their statistics are used by classifiers to define their part values. These part values are then compared with the classification category to detect objects^16^. Later with the introduction of machine learning algorithms in computer vision, object detection from images took a new direction. The key features of objects are extracted to compare and detect objects^17^, and human face detection based on examples^18^ was studied. DL techniques integrated with CNN have been used in image object detection. Some of them used conventional DNN, Regional-CNN, FCNN and so on.

### Video object detection

The object detection algorithms used in images are extended to be used in videos. Video-based object detection algorithms select the key frames from the sequence of frames which will be fed to the DNN for object detection. Instead of just relying on the frame information, the temporal and contextual data in the video is effectively used in detecting the object^19^. A specific category of salient object detection was expanded that aims to detect the regions in the video frame that are potentially possible areas to contain the object^20^. To have an improved detection rate, the CNN models are trained with target images and then used to detect target objects from the video^21^.

Region-based CNN (R-CNN) algorithms are helpful to CNN. They divide the frame or image into regions called region proposals. The region proposal are produced based on the presence of objects in these regions. These region proposals are then fed to the CNN for feature extraction. These R-CNNs are modified in various forms to suit the needs. R-CNN results are analysed to find the statistical differences between the region proposals and bounding boxes defined by CNN to have the object detection path^22^. R-CNN is used in various use cases like real-time surveillance monitoring^22–24^, geospatial video monitoring^25^, medical diagnosis^20,26^ and so on. The other modified forms of R-CNN are F-RCNN (Faster version) and M-RCNN (Mask version). Faster R-CNN has two neural networks: The first CNN calculates the feature map of the frames and this feature frame is used by F-RCNN to locate the object in the frame precisely. Though it has two different neural networks, their performance results are highly appreciable. Faster R-CNN is deployed in object detection and classification^27–30^, combined with other faster algorithms like YOLO^31,32^, cascaded with classifier fusion^33^, and included with key points from the local frame^27^. The Faster R-CNN is improved in many ways by including contextual information from video^34^ and by the use of optimization algorithms in selecting the features and regions^35–37^.

YOLO algorithms are based on a convolutional network with fully connected layers at the end for faster and improved results. YOLO algorithms are known for their easier implementation, faster processing speed and high accuracy^14,38^. Hence, these YOLO algorithms of various versions are used in real-time video monitoring^39^, they are combined with other object detection algorithms like Single Shot Detection (SSD)^40^, making them lightweight for use in energy-restricted devices^41,42^. The YOLO-R algorithm uses two different knowledges namely implicit knowledge and explicit knowledge. The use of explicit knowledge helps YOLO-R in achieving better accuracy compared to conventional YOLO^43^. YOLO-R are shown to have potential benefits in smaller systems^44,45^. Most of the uses of YOLO-R were aimed at the detection and classification of objects in diverse fields^46–48^.

### Traffic surveillance

Traffic monitoring and surveillance is much needed task in areas of heavy traffic. There are multiple ways to monitor the road traffic. Some of the conventional methods include the use of sensors. Sensors are placed at multiple locations to detect the movements and estimate the number of vehicles passing through^49,50^. The sensors were integrated into Wireless Sensor Networks to monitor road traffic for larger areas with inclusive features like phase time optimization^51^, adaptive controlling of traffic lights^52^, and controlling of dynamic traffic^53,54^.

Later with computer vision and deep learning, the traffic videos were monitored in real time. All these techniques were aimed at analysing the traffic video for vehicle detection and classifying the vehicles based on trained labels. Neural networks in the form of CNN, and deep neural networks began to emerge. Spatiotemporal data of the traffic flow is captured and analysed by the recurrent neural networks to get a picture of real-time traffic^55^. DL models with multitask learning capability were able to predict the traffic flow promptly^56^. The FNN network output parameters were optimized using SA optimization to improve the accuracy of traffic vehicle prediction^57^. Some of the other neural network models used in traffic flow prediction were feature extraction with temporal data^58^, the tree-RNN model^58^, the use of Software Defined Network (SDN) in the prediction of traffic^59^ and the use of multimedia data to classify the traffic vehicles^60^.

The volume of traffic is identified by using various forms of YOLO algorithms^61,62^, SORT algorithms^61^, deep neural networks^63^, and integrating vehicular networks with CNN^64^. Traffic surveillance also requires the volume of current traffic on the road to be controlled dynamically. By utilizing algorithms based on object detection, vehicles are classified and the number of vehicles of each class can be found in a location. The use of a neural network in calculating the vehicle count is optimized by the use of optimization algorithms like oppositional gravitational search^65^. To further improvise the prediction, the spatial-temporal data of the vehicles^66,67,78^, the use of graph-based CNN^68–70^, and faster CNN^71^ were researched. These ideas were used to get the current traffic flow and these results were left with the traffic controlling system.

### Overview of methods used in literature

While the above studies highlight the progress of object detection that is elaborated from traditional to deep learning approach, each method has different benefits and limitations^73,77^. Traditional algorithms are computationally effective and easy to use but they are unable to normalize well in dynamic environments such as traffic monitoring^74–76^. Machine learning methodologies improve accuracy and adaptability, but require a broad range dataset and are sensitive to variation in light and view point. Deep learning techniques including fast R-CNN, SSD and Yolo provide advanced accuracy and real-time performance, but their higher computational requirements make them less suitable for object detection with limited resources. Similarly, integration of object detection with edge computing reduces delays and supports real -time decisions making. But this has the limitations such as memory constraint, storage and energy efficiency. These trade-offs emphasize the requirement for optimized solutions that balance accuracy, which is the motivation of the current study.

### Problem statement

Several studies have employed sensor networks to monitor traffic flow, where sensors can effectively capture vehicle counts. However, these approaches often lack advanced processing capabilities, rendering the overall system limited in functionality. Other works have leveraged neural networks for vehicle surveillance on cloud servers, but transmitting traffic videos to the cloud introduces significant latency, which delays traffic control decisions. Edge-layer implementations have also been explored, yet their outcomes have not been effectively integrated into real-time traffic management. Building on these limitations, the present study aims to design and implement a faster and more accurate vehicle classification algorithm on the edge layer, coupled with a cloud-based decision-making system, to enhance the efficiency and responsiveness of intelligent traffic control.

## Materials

### Dataset and methodologies

The dataset used for the proposed method analysis is the Roboflow vehicle image dataset. The high-resolution images are collected using a drone platform. The imagery in this dataset is captured with a wide variety of flight heights, weather conditions, camera angles, time, and illumination. The vehicle-count-in-drone-video dataset is an open-source dataset which was available in the URL https://universe.roboflow.com/altz-bs642/vehicle-count-in-drone-video and published in the Roboflow universe journal during Jan 2023. It consists of 700 images which includes 636 images in the training set, 20 images in the validation set and 44 images in the testing set. In the dataset, the pre-processing is done by applying auto-orientation techniques for resizing the images into 640 × 640 from the original size of 1920 × 1080 and arranged as 2 rows X 2 columns. The sample images are shown in Fig. 4. Images are illuminated in various conditions, including daytime with adequate lighting, night time with insufficient lighting, cloudy, high-intensity light, and glare. The object types are mapped into 9 classes: 2 and 3-wheelers, cars, buses, Hatchback, Sedan, SUVs, Tractors, and Light and heavy commercial vehicles.

The vehicle images were collected from real roads by the drones near the traffic signal. The drone will provide the video surveillance input for our datasets for the proposed implementation. These videos have enough resolution to monitor vehicular movements. To ensure that machine learning models trained on it can generalise successfully to varied situations, the dataset probably comprises a wide range of traffic conditions. This variety could include distinct weather patterns (sunny, cloudy, rainy), different times of day (day, night), different kinds of routes (country, urban), and varying densities of traffic. The ensemble deep learning framework is used in the proposed methodology for object detection and traffic video analytics. The performance metrics used to evaluate the proposed approach are accuracy, precision, recall, F1score, and frames per second. For object detection, tiny YOLO with DEEP YOLOR is used. YOLOv4 outperforms in terms of object detection accuracy and achieving higher performance. YOLOv4 attains greater recall and precision rates by utilising a variety of training techniques and architectural enhancements. YOLOv4 is a dynamic model that can identify many different types of objects in diverse environment settings like traffic video analytics. It is appropriate for a variety of real-world applications due to its ability to identify objects of varying sizes, orientations, and forms and this is the greater requirement for real-time video traffic analytics. By utilizing the potential feature fusion methodology, YOLO v4 can be implemented with various resolutions and sizes, which in turn will produce more accurate identification of vehicles of different sizes.

### Ensemble learning in traffic video analytics

Ensemble models are identified as one of the most existing potential techniques in traffic video analytics, since they are good in achieving high prediction accuracy and robustness by integrating the approached from multiple models. The goal of employing ensemble models is to lower the prediction’s generalization error. Using the ensemble approach reduces the model’s prediction error provided that the base models are independent and diversified. The method bases its forecast on the wisdom of the multitude. The ensemble model functions and behaves as a single model even when it has several foundation models. The majority of real-world data mining solutions make use of ensemble modelling methods. The strength ensemble model in traffic video analytics lies in its ability to combine multi-scale feature extraction with attention mechanisms, capable of capturing detailed information about small and large objects in a scene This adaptability is important for traffic video analysis, including viewpoint distortion, occlusion, differentiation.

### Data preprocessing techniques

The primary phase of data pre-processing operation is removing the noise. This step involves the elimination of any noise or visual artefacts, such as motion blur, sensor noise, or lens distortion. The purpose of this step is to: (i) improve the quality of the images by removing periodic or random patterns using filters; and (ii) enhance the images to highlight and improve the features of the lesions that will be used to train the DNN. The photos are processed to (i) reduce the noise produced during fundus image capture, (ii) fix lighting irregularities, and (iii) enhance and improve the contrast of the photographs. A 5 × 5 median filter was employed to smooth the image. The non-linear median filter was employed to eliminate impulsive noise, or large-amplitude irregular pulses, like those found in Salt &Pepper. An image is divided into contextual sections, or tiles, using the CLAHE algorithm. Every contextual region’s histogram is produced, and clipping is done at a predetermined value. The histogram bins receive a redistribution of the clipped amount. The original histogram has been adjusted to create this one. This technique lessens the issue of over-enhancement and eliminates the edge-shadowing effect of AHE. The effectiveness of CLAHE has been shown in improving low-contrast medical images. The size of the contextual zone and the histogram’s clip limit are the two criteria that need to be considered for CLAHE. The CLAHE outcome may be impacted by these parameters. By redistributing the employed grey levels, this technique enhances the visibility of the image’s hidden features. An image’s local contrast should be maintained since it influences the visual contrast overall. The condition helps in preserving local contrast are

1 $$\:\frac{O(i,j)}{{O}_{avg\:}(i,j)}=\frac{I(i,j)}{{l}_{avg\:}(i,j)}$$

Where $\:O(i,j),I(i,j)$ denotes the output luminance and input luminance level and $\:{O}_{avg\:}(i,j),{I}_{avg\:}(i,j)$ denotes the output local average and input local average.

The fundamental equation denotes the requirement to maintain the local contrast during the dynamic range compression procedure is denoted as

2 $$\:O(i,j)=p(/(i,j\left)\right)\times\:r\left(/(i,j),{I}_{avg\:}(i,j)\right)$$

The enhanced image after this pre-processing step is used for effective object detection using tiny YOLO and deep YOLOR.

### Proposed methodology

**Fig. 2:**
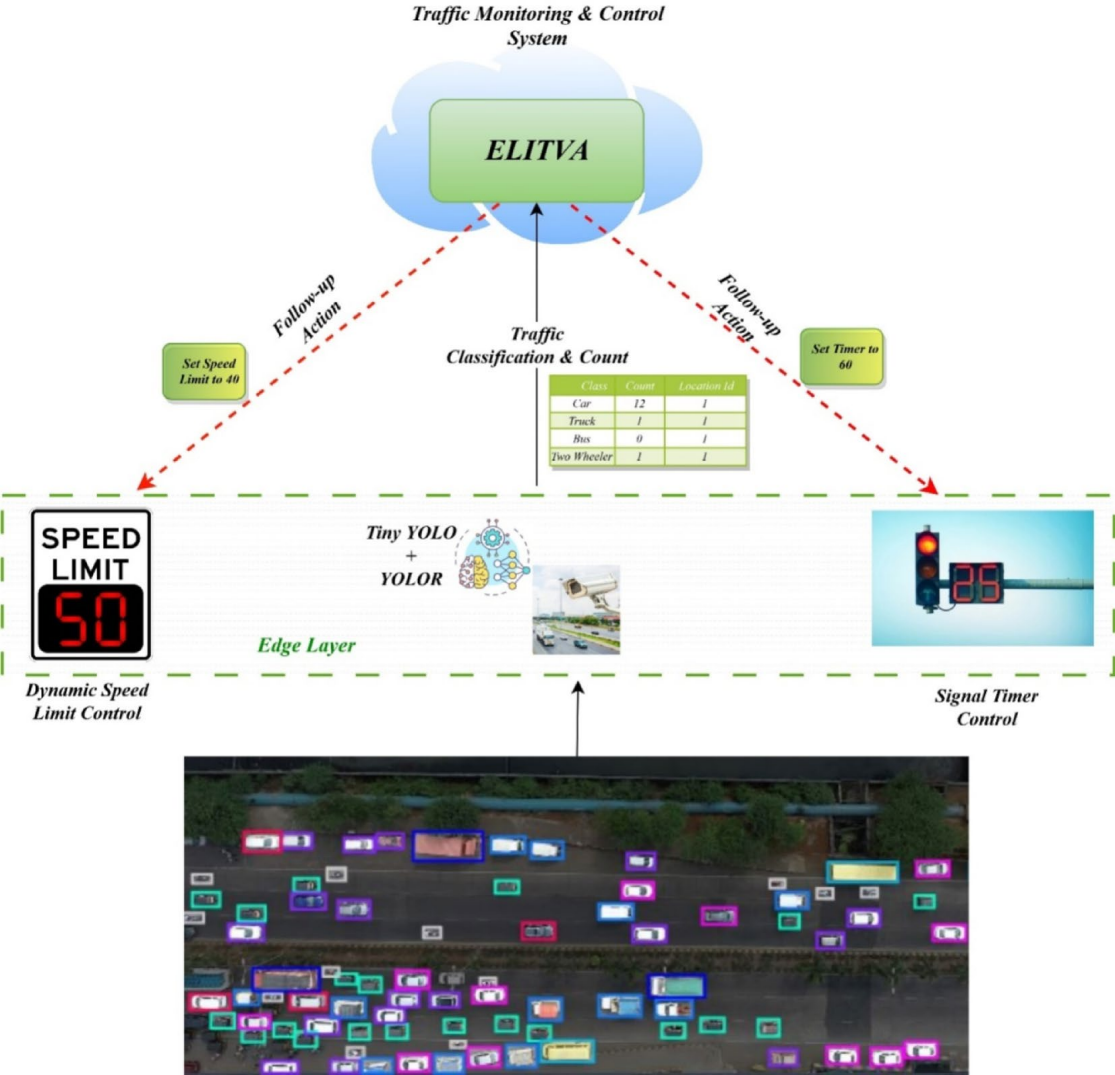
Architecture of Proposed ELITVA Model.

One of the common and difficult issues in computer vision is object detection. Utilizing underlying deep mathematical models, authors have experimented extensively and contributed to the efficiency rise for object identification and related tasks including object classification, localization, and segmentation during the past ten years due to the rapid evolution of deep learning. The proposed model ELITVA uses the ensemble deep learning approach that uses a local model and a global model. The proposed flow is shown in Fig. 2. The local model uses the Tiny YOLO and Deep YOLOR in the edge server whereas the global model uses a Fast Recurrent Neural Network (FRNN). Object detection is done by YOLO models in the edge layer which detect the vehicles of various categories. The video analytical information is retrieved in the cloud layer by FRNN and the same is sent to the control room for further actions.

### Tiny YOLO in edge layer

The object identification technique, which employs deep learning, mimics the tasks required for object prediction in images, thereby lowering computational complexity.

A light version YOLO (you only see once) family of object detection algorithms is the Tiny YOLO. It has been widely used for real -time applications due to speed, its lower calculation complexity and rapid inference speed. Unlike full YOLO models, which require powerful GPUs, tiny Yolo is designed with less specific layers, making it suitable for distribution on resources such as edge nodes and embedded systems. However, this efficiency falls at the expense of low detection accuracy, especially in cases associated with small or overlapping objects. Despite this limitation, Tiny Yolo is the most promising algorithms for traffic monitoring, as it provides a practical trade-off between accuracy and speed. In the current study, Tiny Yolo acts as the basis for developing a customized vehicle classification model that balances the performance with hardware constraints on the edge equipment. CNN helps in capturing repeating patterns in multidimensional data fields. For object detection in deep learning systems, convolutional neural networks are commonly used. This algorithm is given an input image, divides it into classes using learnable weights and biases, and then applies the results to the image. The YOLO algorithm will scan the whole image, and detect it with the help of all its information. In contrast to Regional Convolution Neural Network Detectors (R-CNND), which require more space for a single image, YOLO uses a single network assessment. Every region proposal in R-CNN is categorized and improved separately. This includes:

- Area of Concern^33^: Pooling is the process of warping features from various regions to a standard size.
- Classifier Head: A classifier to forecast the class label, which comprises of SoftMax with fully connected layer.
- Bounding box regressor: An additional output layer called the Bounding Box Regressor modifies the bounding box dimensions.

**Fig. 3:**
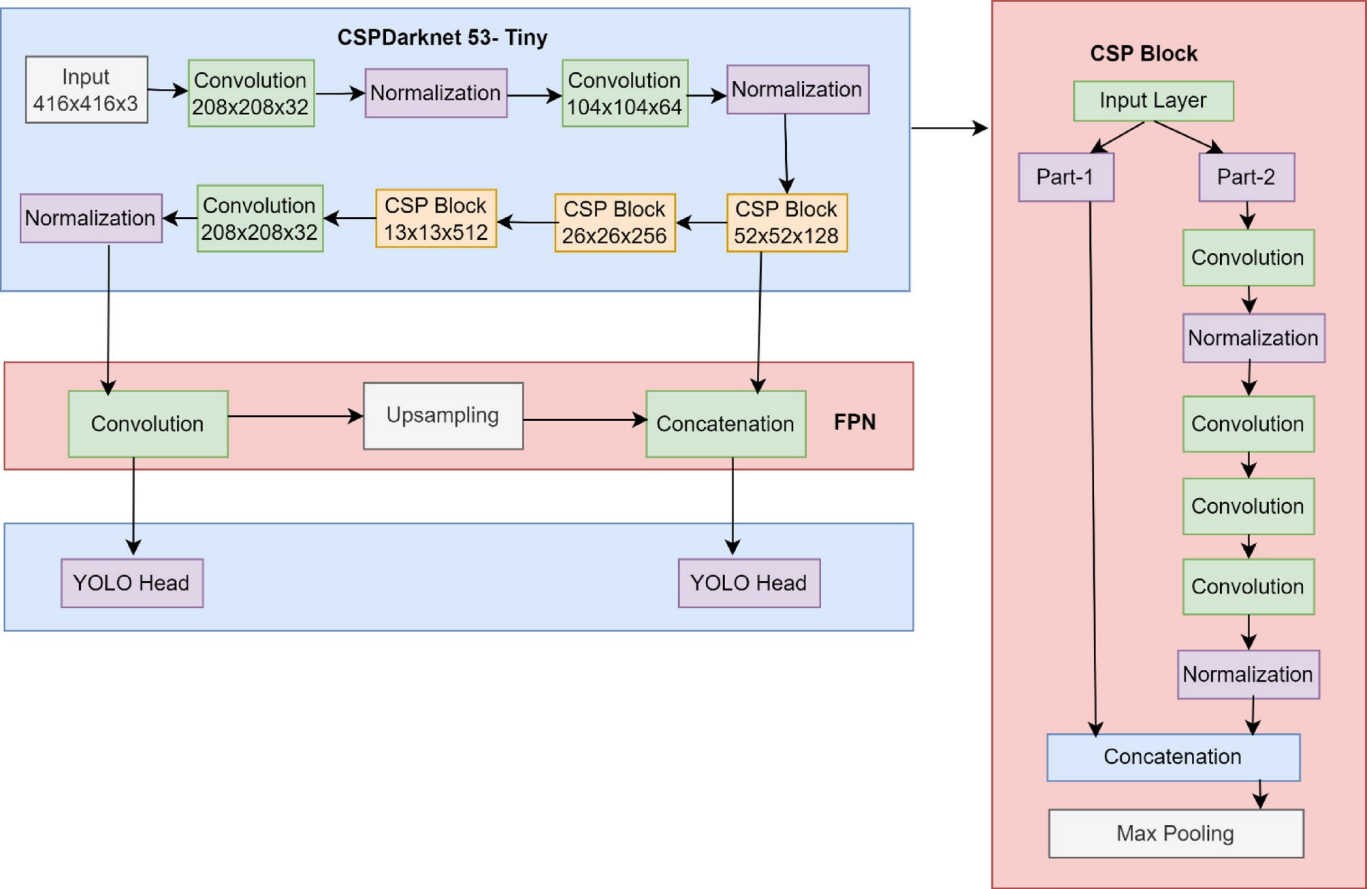
Architecture of YOLOV4-Tiny.

To maximize both classification accuracy and bounding box precision, the model is trained using a combination of regression loss such as smooth L1 loss and classification loss such as SoftMax loss. After inference, the model refines the bounding boxes and eliminates redundant detections by using Non-Maximum Suppression (NMS). The image is first split up into several areas or proposals that probably include things. These ideas are produced using conventional techniques such as Edge Boxes and Selective Search. The model collects and divides input characteristics of an image into an S × S grid. The proposed approach makes use of the YOLOv4-tiny for faster object recognition in real-time traffic. The architecture of YOLOv4-Tiny is specified in Fig. 3. YOLOv4-Tiny uses a variety of modifications from the original YOLOv4 model to maximize the output on consumer electronic devices. Initially, the CSP backbone has a reduced number of convolutional layers. In addition, there are now two YOLO layers rather than three, as well as a modest number of prediction anchor boxes.

This consists of three layers, namely CSPDarknet53- Tiny, Feature Pyramid Network (FPN), and YOLOHead. The core feature extraction approach employs CSPDarknet53-Tiny, which is made up of CSPBlock and Conv block. This layer includes activation functions as well as batch normalisation. Model regulation is performed using batch normalisation. It replaced the need to employ the architecture’s dropout layers to address overfitting concerns. The process of fine tuning the variance will improve the normalization of the input. The activation function in the proposed approach is leaky ReLu (Rectified Linear Unit) functions. CSPBlock separates the base layer model into a pair of pieces. The initial component is developed as a residual edge, and the second is combined with the initial layer to create the final output is generated after passing a number of convolutional methods. An FPN structure can combine the attributes of several network layers, saving low-level network geometric information as well as deep network semantic data. The final product is known as a dense prediction, and it comprises a vector output that holds the predicted bounding box coordinates (height, width, and centre), the prediction’s label, and the confidence score. Non-Maximum Suppression (NMS) is an important tactic in YOLO which uses Intersection over Union (IoU) threshold of **0.5** and a confidence score cutoff of **0.3** for filtering detections. The steps involved in NMS are shown in Algorithm. 1. It starts by ranking each detection box based on its score.

**Algorithm. 1:**
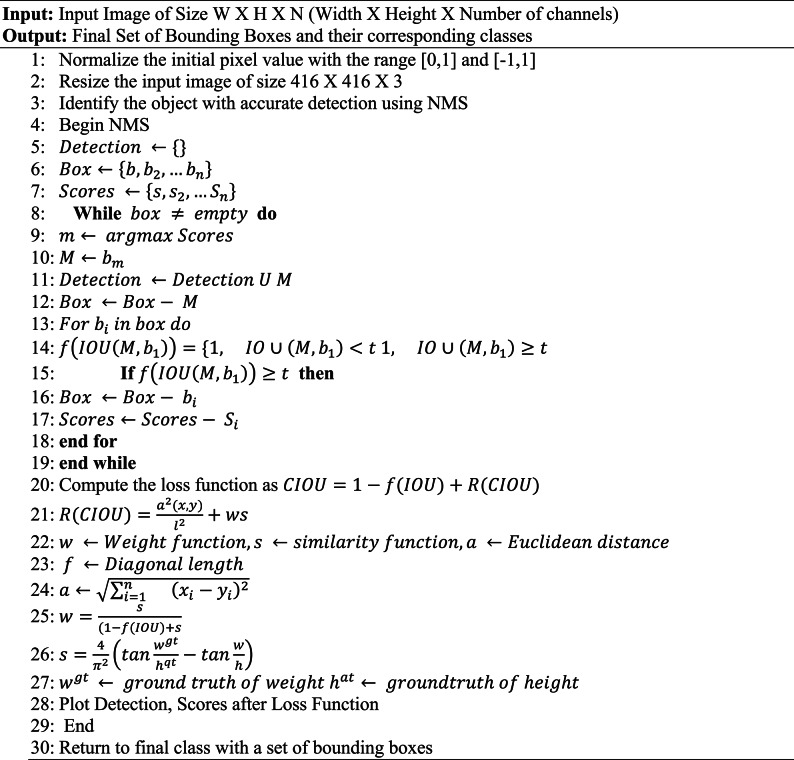
Object Detection using ELITVA.

All other detection boxes that have a considerable overlap based on a predetermined threshold with detection box M are suppressed, leaving only the detection box M with the highest score. Recursively, the same procedure is applied to the remaining boxes. The IOU of the box with the highest score above a predetermined threshold is immediately set to zero by the NMS. Numerous automobiles are overlapping while being monitored by traffic cameras. To cope with overlapping cars, we employ soft-NMS instead of NMS for post-processing, screening out inaccurate regions. The classic greedy NMS method is modified by Soft-NMS such that, as opposed to NMS, we overlap and do not set the score to zero. The Billion Float Operations Per Second (BFLOPS) implies the number of floating-point operations involved in CNN. It is calculated as shown in Eq. 3.

3 $$\:BLOPS=\frac{2HW{K}_{i}{K}_{j}{C}_{i}{C}_{o}}{{10}^{9}}$$

Where $\:{K}_{i}{K}_{j}$ denotes the Kernel size and $\:{C}_{i}{C}_{o}\:$denotes the number of channels in the Input and Output layer.

### Deep YOLOR model

Deep YOLOR (You Only Learn One Representation) represents a major breakthrough in traffic video analysis by combining object recognition and position learning capabilities in a system. This innovative approach allows the recognition of various vehicle objects accidentally in real time, highly accurate and classified, such as vehicle prediction, pedestrian and traffic signs identification. Deep YOLOR can process large amounts of video data efficiently, detect and track objects accurately it is remarkable it is particularly useful in complex urban environments where accurate detection of fast-moving objects is critical for effective traffic management and safety maintenance. YOLOR is an advanced object detection model. YOLOR initially pre-trains the implicit knowledge model with a series of tasks from the existing dataset. Deep YOLOR trains an additional number of attributes that specify explicit knowledge. Both implicit and explicit information are used for the final prediction. By reducing the vanishing gradient issue, residual connections made popular by ResNet architectures allow deeper networks to be trained more successfully. Deep neural networks, which are capable of capturing more intricate aspects and representations, can be trained as a result. Objects of interest can vary greatly in size and appearance due to different lighting conditions. The Deep YOLOR architecture also incorporates advanced techniques such as spatial pyramid pooling and path aggregation, providing the ability to detect objects at different scales in is greater, composite model -And improve efficiency Consequently, traffic management systems can use accurate vehicle density, speed estimation and incident detection, resulting in smooth traffic flow and reduced by accident. Moreover, Deep YOLOR can constantly enhance its performance over time due to its capacity to learn from and adapt to large datasets. This capacity for self-learning is especially crucial for traffic video analytics, as patterns and behaviours in traffic might vary as a result of infrastructure modifications, seasonal fluctuations, and new developments in urban transportation. Traffic authorities and smart city planners can apply data-driven solutions for traffic optimization and urban planning by using Deep YOLOR, which provides them with deeper insights into traffic dynamics. The proposed methodology can outperform current deep learning approaches in traffic video analysis due to its unique combination of object detection and position learning in one simplified framework. Unlike traditional models which often require separate steps each for object extraction, detection and classification, Deep YOLOR combines these techniques, making it more efficient and data binding. This combination reduces latency and increases real-time processing capabilities, of which needed in traffic video analysis where timely and accurate information is critical for decision-making. Furthermore, Deep YOLOR incorporating multi-scale feature extraction and attention devices of different sizes, organizes and visualizes objects and tracking thereby solving common traffic crash challenges.

- *Processing of Input Image*: A grid is created from the image.
- *Prediction*: Multiple bounding boxes, along with the confidence scores and probability classes that relate to them, are predicted for each grid cell.
- *Non-Maximum Suppression*: A subsequent processing stage to improve bounding boxes and get rid of duplicate detections.

**Fig. 4:**
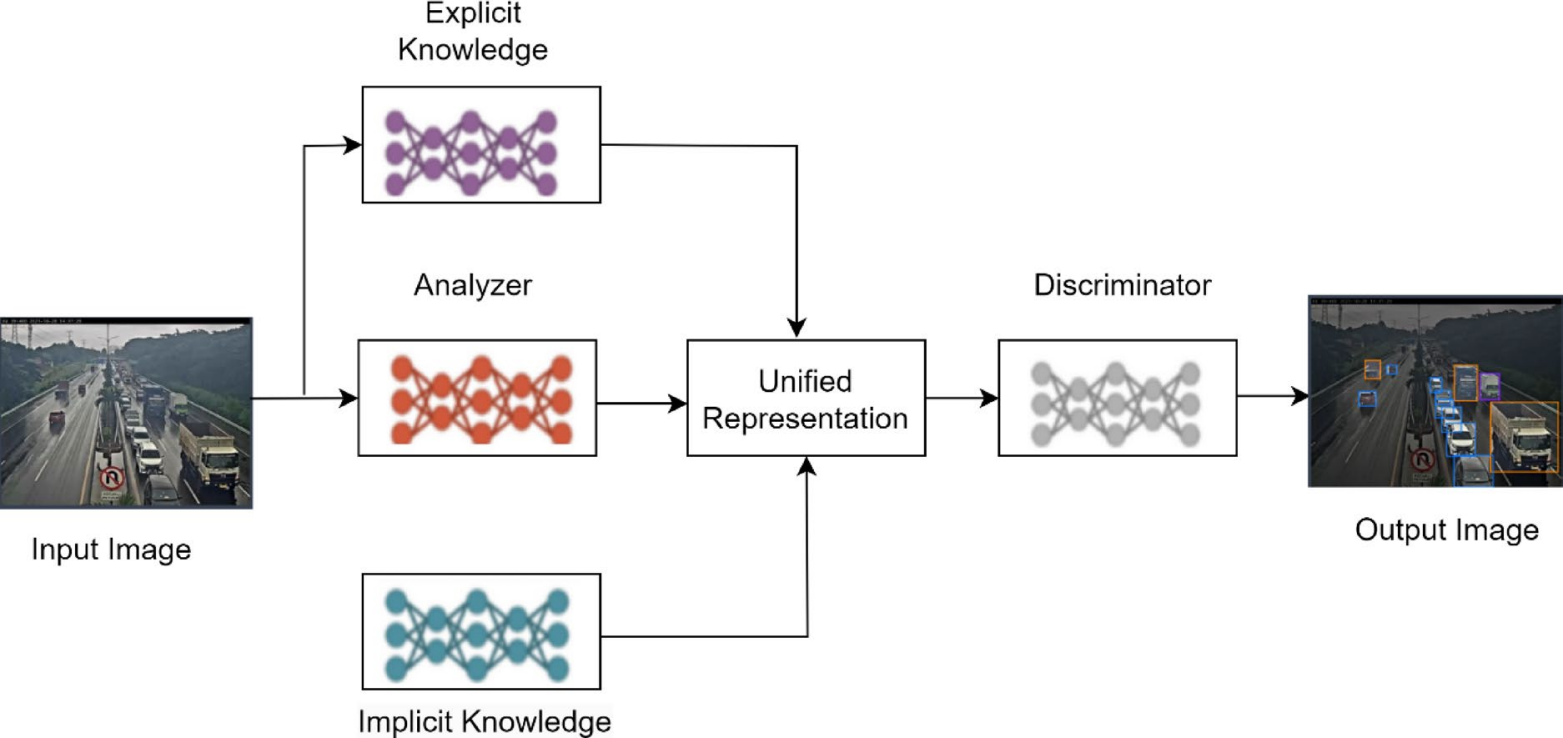
Architecture of deep YOLOR.

The incorporation of residual connections into Deep YOLOR probably improves the model’s performance and training, particularly when working with extremely deep architectures. The architecture of Deep YOLOR is depicted in Figure. 4. Explicit knowledge refers to any data that can be expressed in writing and comprehended in a way that allows specific conclusions to be formed.

These data are fact-based and might be obtained via shallow neural networks, while conscious learning produces explicit knowledge. Implicit DL consists of two basic components: deep equilibrium models and implicit neural representations. The former essentially accomplishes various tasks by acquiring the parameterized continuous mapping representation of discrete inputs, whilst the latter converts implicit learning into residual form neural networks and computes the equilibrium point on them. Kernel space misalignment is a typical problem in neural networks with several tasks and heads.In order to solve this issue, we can multiply and add output features as well as implicit representations. This allows us to translate, rotate, and scale kernel space to align each neural network’s output kernel space. The conventional neural network is expressed in Eq. 4.

4 $$\:y={f}_{\theta\:}\left(x\right)+\delta\:$$

Where Xis the observation, $\theta$ are the neural network parameters, $\delta$ is the error term and Y is the target function. In the training process of the implicit DL model, $f_{\theta}(x)$ must be close to the target function.We use explicit and implicit information to model the error term, which is then used to direct the multi-purpose network training procedure to train the suggested unified networks. The training equivalent equation is as follows.

5 $$\:y={f}_{\theta\:}\left(x\right)+\delta\:+{g}_{\theta\:}\left({\delta\:}_{i}\left(x\right)+{\delta\:}_{e}\left(x\right)\right)$$

Where ${\delta}_{i}(x),{\delta}_{e}(x)$ are the implicit and explicit errors from the observation X,$g_{\theta}(s)$ is the task-specific unified representation of an explicit and implicit representation. The error terms are extended to include the following representation as follows.

6 $$\:T(x,\theta\:,S,Y,\psi\:)=0$$

Here $[S = {Z_1},{Z_2},{Z_3}....{Z_n}]$ is the latent code of implicit learning and $\psi$ is used to generate the output of output parameters. To converge the error terms to very minimal the following functions can be included as shown in Eq. 7.

7 $$\:{d}_{\psi\:}=\left({f}_{\theta\:}\left(x\right),{g}_{\theta\:}\left(z\right),y\right)=0$$

For the different tasks in the unified representation, the process initially starts with the $f_{\theta}(x)$ and converges to the implicit DL representations $g_{\theta}(z)$ and computes the final output with the discriminator$d_{\psi}$ .

### Fast recurrent neural network (FRNN) for video analytics

The object is detected with the edge layer using the aforementioned model. The output from the object detection and analytical information is given as input to FRNN. RNN is a family of ANN that are used to interpret words, temperatures, and daily stock prices, among other information sequences. These algorithms have no fixed size limit and are made to accept a sequence of inputs. RNN consists of three layers such as (a) Input Layer (b) Hidden Layer and (c) Output Layer. The number of nodes in the input layer of an RNN is always determined by the dimension of the data. These nodes are connected to the hidden layer by connections known as weight, a coefficient found in the relationship between each pair of nodes from the input to the hidden layer. It serves as a signal decision-maker. Naturally, learning involves constant weight adjustments after learning is complete. Because video data is sequential, recurrent neural networks (RNNs) might be computationally costly for video analytics applications like action recognition, video captioning, or video summarizing. They can be faster than conventional RNNs for some applications because they use dilated convolutions to capture long-range relationships in the input sequence while preserving parallelization. Convolutional layers can be used to make use of the advantages of either recurrent or attention methods. Convolutional layers, for example, can be used to extract features and recurrent or attention-based models can be used to model sequences and make decisions. The RNN will have the ideal weights for individual synapses. The activation function also defined as sum of weights which is initiated from the input layer, is applied as a sigmoid or tangent hyperbolic (tanh) function by the hidden layer nodes. This operational step will generate values with minimized error rate among the train and test data using SoftMax function. The output layer of the proposed method is generated based on the values from these layers. The backpropagation technique is applied to get the optimal error value at this moment. This connection existing between the output layer and the hidden layer will send a signal based on the weights to produce an optimal error for the chosen number of epochs. Bidirectional LSTM is a special kind of RNN where forward LSTM is denoted by and backward LSTM is denoted by. At every timestamp, RNN produces the hidden state by combining the forward hidden state $\:\overrightarrow{{h}_{st}}$ and the backwards hidden state $\:{{h}_{st}}$ .

8 $$\:\overrightarrow{{h}_{st}}=LSTM\left({i}_{t},\overrightarrow{{h}_{t-1}}\right)$$

9 $$\:{h}_{st}=LSTM\left({i}_{t},{h}_{t+1}\right)$$

An alternative to the conventional RNN is known as a gated recurrent unit network. Lowering the number of trainable parameters in each cell is intended to simplify the intricate architecture of RNN. It has demonstrated that it can perform better than other models now in use. The two gates that are used for utilizing the hidden state are the reset gate$\:r{g}_{t}$ and the update gate$u_t$.

10 $$\:r{g}_{t}=\sigma\:\left({W}_{rg}{i}_{t}+{U}_{rg}{h}_{z-1}+{b}_{rg}\right)$$

11 $$\:{u}_{t}=\sigma\:\left({W}_{u}{i}_{t}+{U}_{u}{h}_{t-1}+{b}_{u}\right)$$

Where $\:{W}_{rg},\:{W}_{u}$ are the weight terms and $\:{b}_{rg},{b}_{u}\:$are the bias terms.

The FRNN formalizes the input data and produces the output which decides the speed limit for the previous signal and normalizes the timer in the successive signal. Thus, the proposed strategy provides better object detection and video analytical information in traffic surveillance.

## Experimentation, results &analysis

### Experimental setup

This section describes the details of all experiments as well as their comparisons. To assess the performance of the proposed approach, ELITVA was executed over drone-acquired videos as datasets. The hybrid model combining Tiny-YOLO and YOLOR for edge computing was applied in the proposed technique. The vehicle-count-in-drone-video dataset is an open-source dataset published in the Roboflow Universe Journal in January 2023 and accessible at https://universe.roboflow.com/altz-bs642/vehicle-count-in-drone-video. It consists of 700 images, of which 636 belong to the training set, 20 to the validation set, and 44 to the testing set. Preprocessing was carried out using auto-orientation techniques to resize the images from their original 1920 × 1080 resolution to 640 × 640, arranged in a 2 × 2 grid. The dataset includes diverse illumination conditions such as daytime, cloudy, high-intensity light, night-time, and glare. The objects are categorized into nine classes: 2- and 3-wheelers, cars, buses, hatchbacks, sedans, SUVs, tractors, and light and heavy commercial vehicles. Sample images are shown in Fig. 4. The experimental environment consisted of a workstation running Ubuntu 20.04 LTS with Python 3.8 scripts and the Roboflow SDK. Model training was performed on a system equipped with an Intel Core i7 CPU (2.6 GHz, 6 cores), 32 GB RAM, and an NVIDIA GeForce RTX 2080 GPU with 8 GB VRAM. For edge deployment, experiments were conducted on the NVIDIA Jetson Xavier NX platform, which features a 6-core ARM v8.2 64-bit CPU, 384-core Volta GPU with 48 Tensor Cores, and 8 GB LPDDR4x memory, designed specifically for AI applications at the edge.

The hyperparameters used during training, validation, and testing were kept consistent across experiments. The training was carried out for 180 epochs with an input image resolution of 640 × 640. To ensure reproducibility and transparency in the training process, the following settings were applied:

- Batch size: 16.
- Learning rate: 0.001.
- Optimizer: Adam optimizer.
- Validation strategy: Stratified split with an 80-10-10 ratio for training, validation, and testing.

The results of the proposed ELITVA model were compared with YOLO-CFNN, YOLOv4, and YOLOv4-tiny. For fairness, the existing data and parameter setups of these models were adopted from the respective cited works, as summarized in Table 1.

**Table 1:** Comparison of parameters setup of various YOLO models.

Model	Batch Size	Learning Rate	Optimizer	Epochs	Input Size
YOLO-CFNN^72^	32	0.001	SGD	100	416 × 416
YOLOv4^72^	64	0.001	Adam	150	416 × 416
YOLOv4-Tiny^72^	64	0.001	SGD	150	416 × 416
**Proposed Model**	**16**	**0.001**	**Adam**	**200**	**416 × 416**

### Experimental parameters

This proposed hybrid architecture with tiny YOLO and YOLOR proposed in Sect. 4 outperforms the existing methods in the literature. The metrics considered for assessing the proposed system are recall, precision, F1 score, accuracy and Frames per second which are explained below with the help of TP which represents the count of genuine positive; FP indicated the count of wrongly chosen positives, TN is used to indicate the count of genuine negative and FN represents the count of wrongly chosen negatives. The model was run 200 epochs using different random seeds to account for variability in training. The reported results represent the mean across these runs. The number of samples per class for training, validation and testing sets are shown in Table 2.

#### Precision

**Fig. 5:**
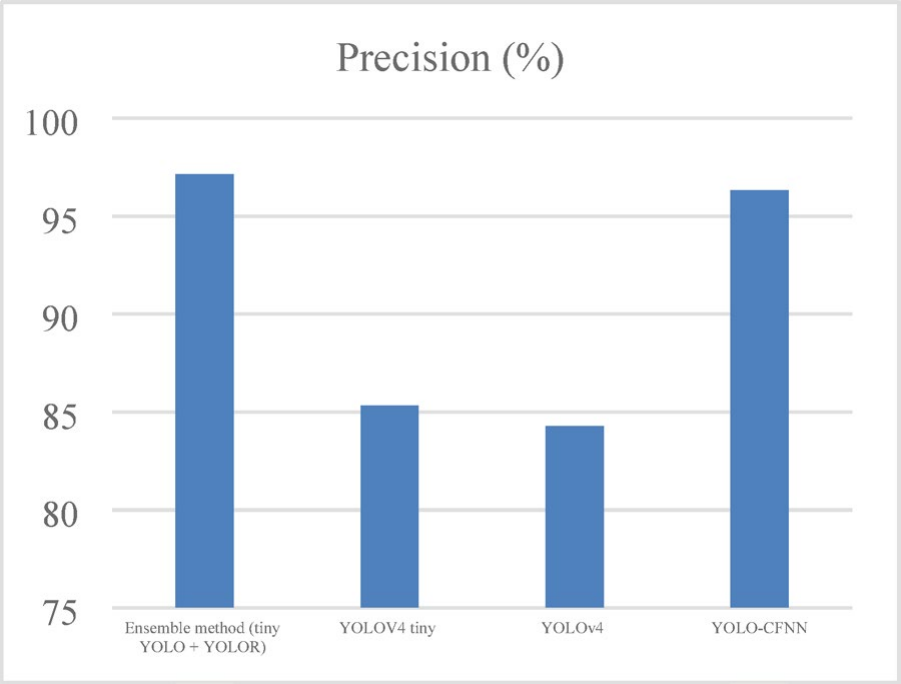
Precision analysis of ELITVA.

Precision is a metric that measures the proportion of genuine positives to the total number of positives predicted by the model as defined in Eq. (12) and shown in Fig. 5.

12 $$\:Precision=\frac{TP}{TP+FP}$$

**Table 2:** Split-up of number of samples per class.

Class	Training samples	Validation aamples	Test samples
2- & 3-Wheelers	120	4	8
Cars	150	5	10
Buses	60	2	4
Hatchbacks	80	2	4
Sedans	70	2	4
SUVs	80	2	4
Tractors	26	1	1
Light Commercial Vehicles	25	1	1
Heavy Commercial Vehicles	25	1	1
**Total**	**636**	**20**	**37**

#### Accuracy

**Fig. 6:**
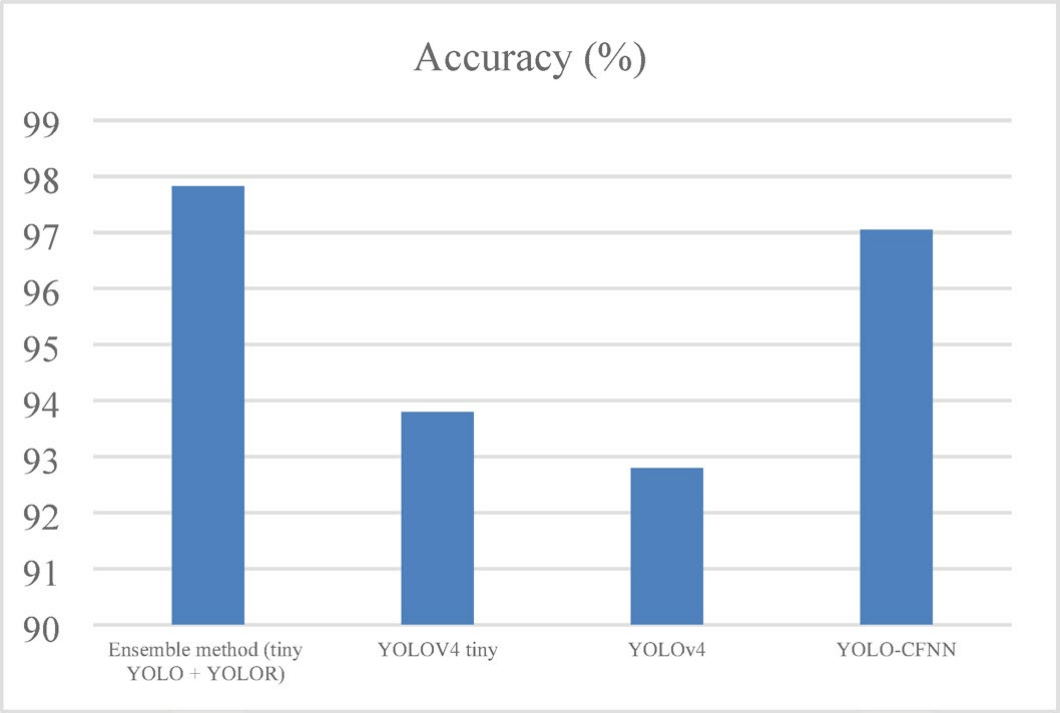
Accuracy analysis of ELITVA.

Accuracy is the most common metric which will return how many were true out of all the predictions considered as shown in Eq. (13) and shown in Fig. 6.

13 $$\:Accuracy\:=\frac{TP+TN}{TP+TN+FP+FN}$$

#### Recall

**Fig. 7:**
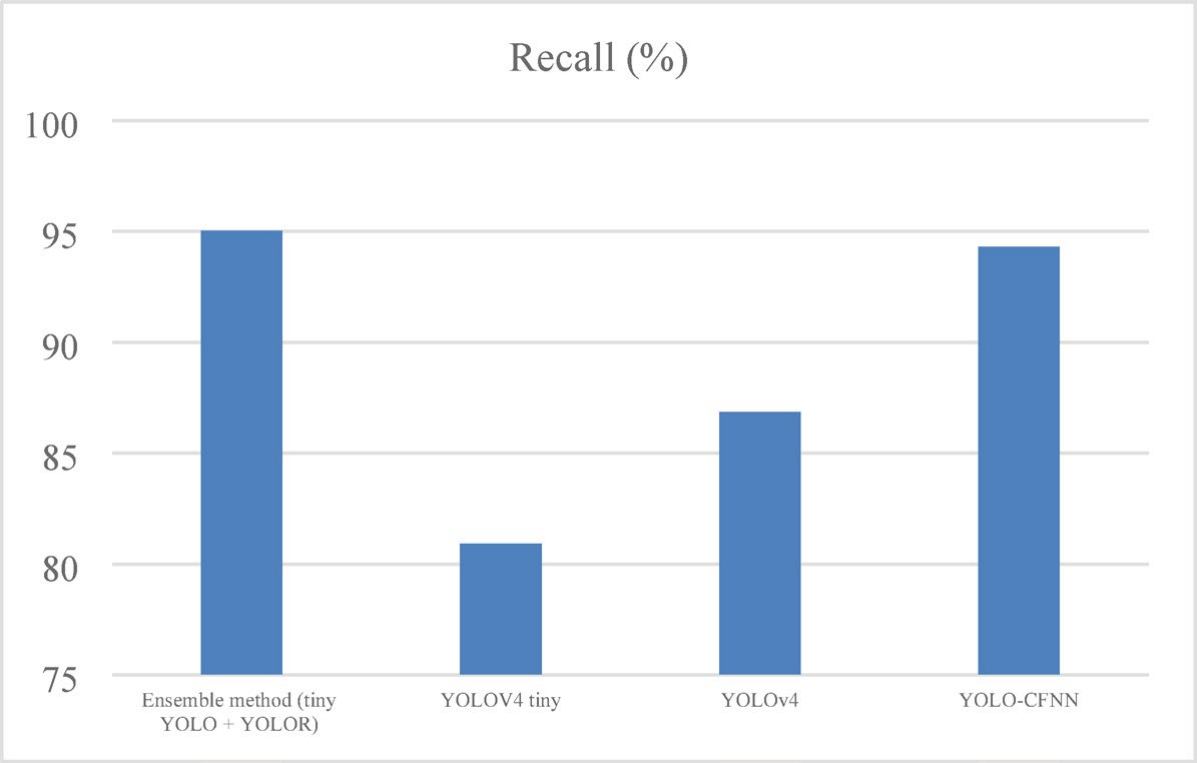
Recall analysis of ELITVA.

Recall focuses on the model’s ability to find all of the positives as shown in Eq. (14) and shown in Fig. 7.

14 $$\:Recall\:=\frac{TP}{TP+FN}$$

#### F1 score

**Fig. 8:**
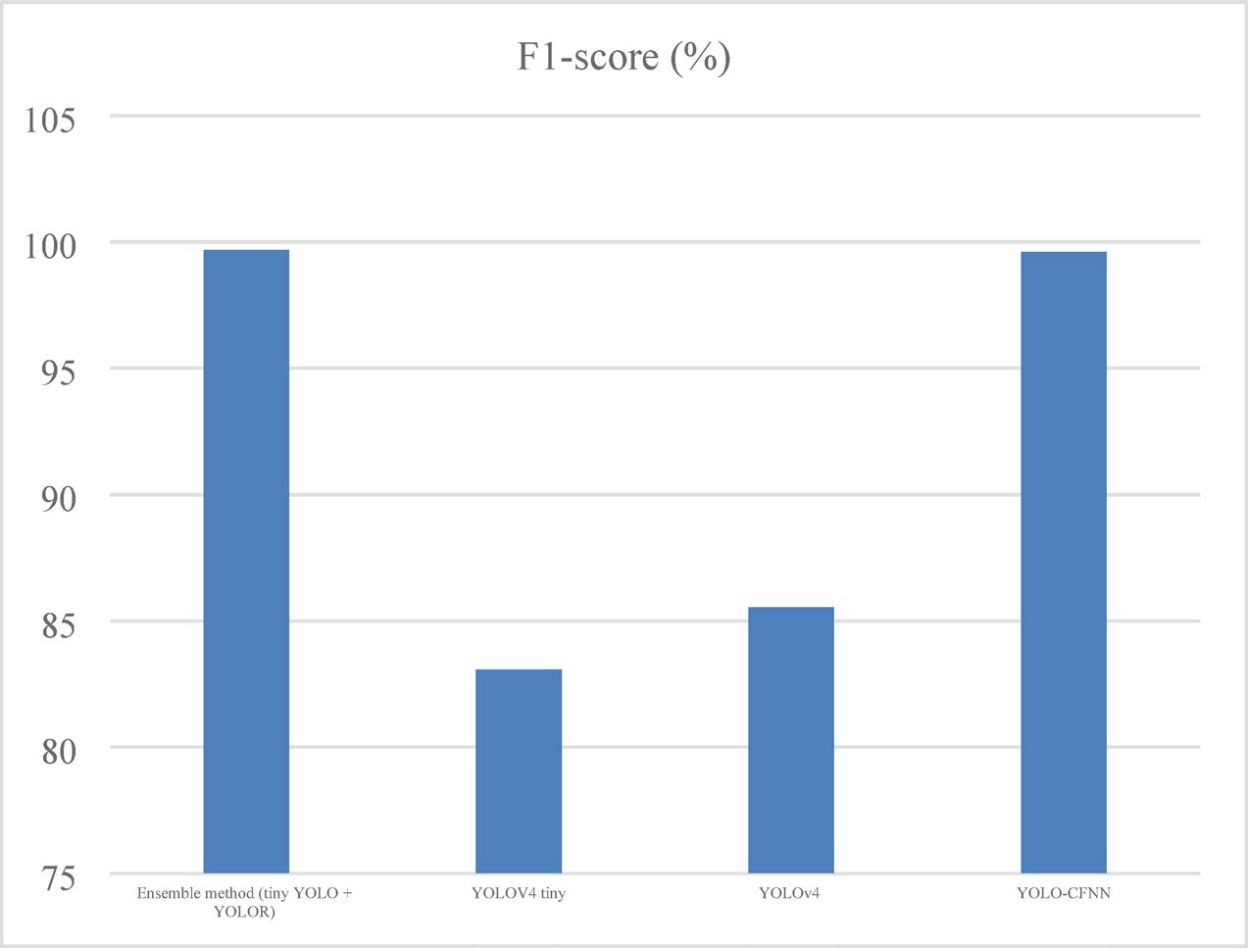
F1-Score analysis of ELITVA.

It is defined as the ratio of twice the product of precision and recall to the summation of precision and recall as given in Eq. (15) and shown in Fig. 8.

15 $$\:F1=2\text{*}\frac{Precision\text{*}recall}{Precision+recall}$$

#### Frames per second analysis

**Fig. 9:**
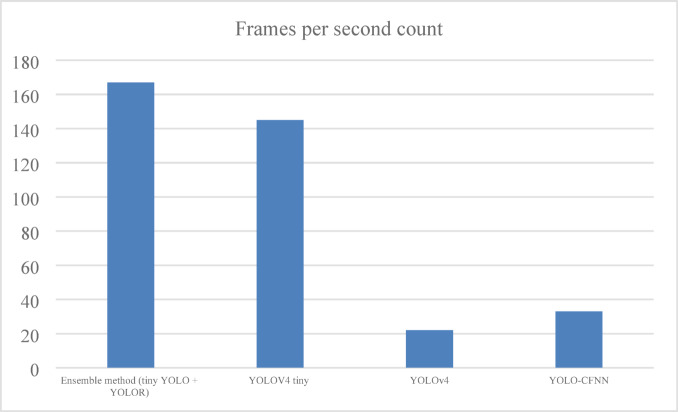
Frames per second analysis of ELITVA.

It is estimated as the ratio of frames to the time (in seconds) as stated in the Eq. (16) and illustrated in Fig. 9.

16 $$\:Frames\:per\:second\:=\frac{Number\:of\:Frames\:}{\:Time\:in\:Sec\:}$$

### Overall analysis of ELITVA

**Fig. 10:**
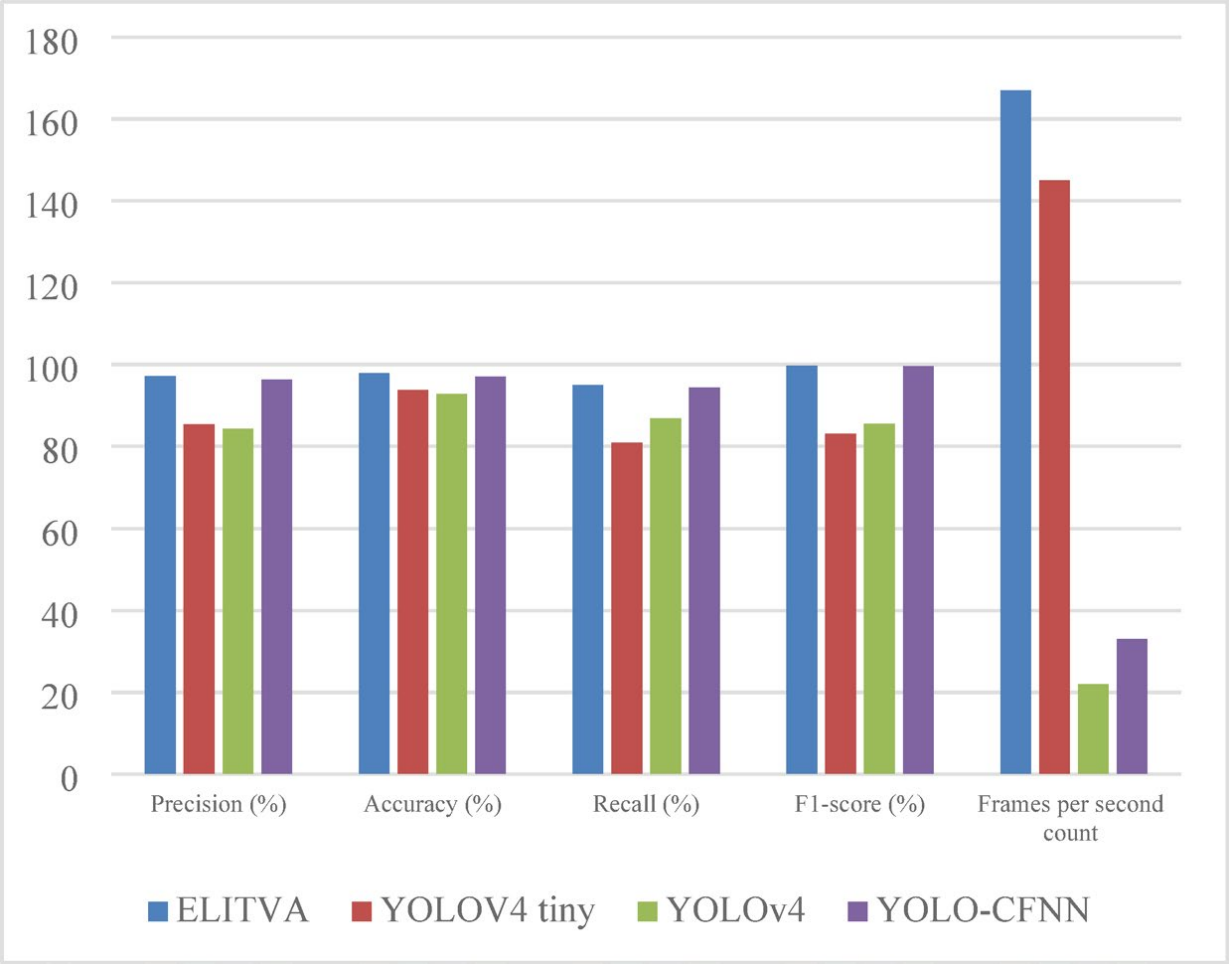
Overall analysis of ELITVA.

The detection performance of ELITVA has been increased by combining tiny-YOLO with YOLOR in the integrated model which is shown in Fig. 10. The suggested architecture for object detection is more accurate after combining tiny-YOLO with YOLOR ensemble learning. In addition, the proposed model employs RESNET as the backbone neural network for mapping the features of the images. The CSP backbone contains fewer convolutional layers. In addition, there are two YOLO layers instead of three, as well as a small number of prediction anchor boxes. This consists of three layers, namely CSPDarknet53- Tiny, Feature Pyramid Network (FPN), and YOLO Head. The basic feature extraction approach makes use of CSPDarknet53-Tiny, which is made up of two blocks: CSP Block and Convolutional Block. Convolutional layers use activation functions as well as batch normalisation to solve overfitting concerns. Table 3 presents a comparative evaluation of the proposed ELITVA model against existing YOLO-based approaches. ELITVA consistently outperformed the other methods across all key performance metrics. Specifically, it achieved a precision of 97.14% and an accuracy of 97.83%, which are higher than those of YOLOv4-tiny (85.33% and 93.80%) and YOLOv4 (84.29% and 92.80%). The recall value of 95.03% further indicates that ELITVA was able to detect the majority of vehicles present, minimizing missed detections. In terms of balanced performance, the F1-score of 96.09% demonstrates its robustness, exceeding YOLO-CFNN’s 95.31%. Importantly, ELITVA attained a frame processing rate of 167 FPS, substantially faster than YOLOv4 (22 FPS) and YOLO-CFNN (33 FPS), and even surpassing YOLOv4-tiny (145 FPS). These results confirm that ELITVA not only delivers superior detection accuracy but also maintains real-time efficiency, making it highly suitable for deployment in edge-based traffic surveillance systems.

**Table 3:** Overall analysis of ELITVA.

Method	Precision [48]	Accuracy [48]	Recall [48]	F1-score [48]	Frames per second count
ELITVA	97.14	97.83	95.03	96.09	167
YOLOV4 tiny^72^	85.33	93.80	80.93	83.07	145
YOLOv4^72^	84.29	92.80	86.86	85.55	22
YOLO-CFNN^72^	96.33	97.05	94.31	95.31	33

**Fig. 11:**
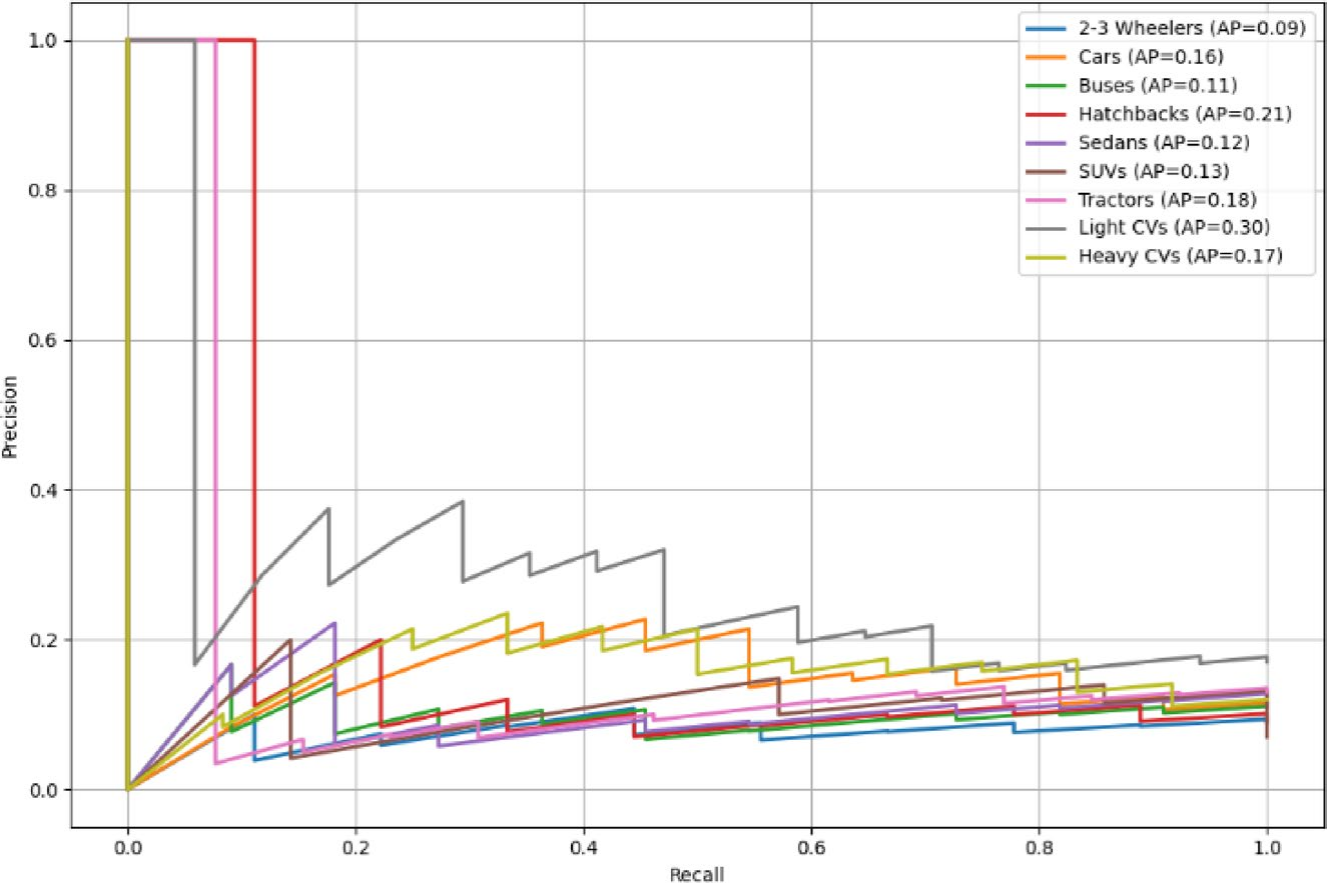
Precision-recall (PR) curve.

**Fig. 12:**
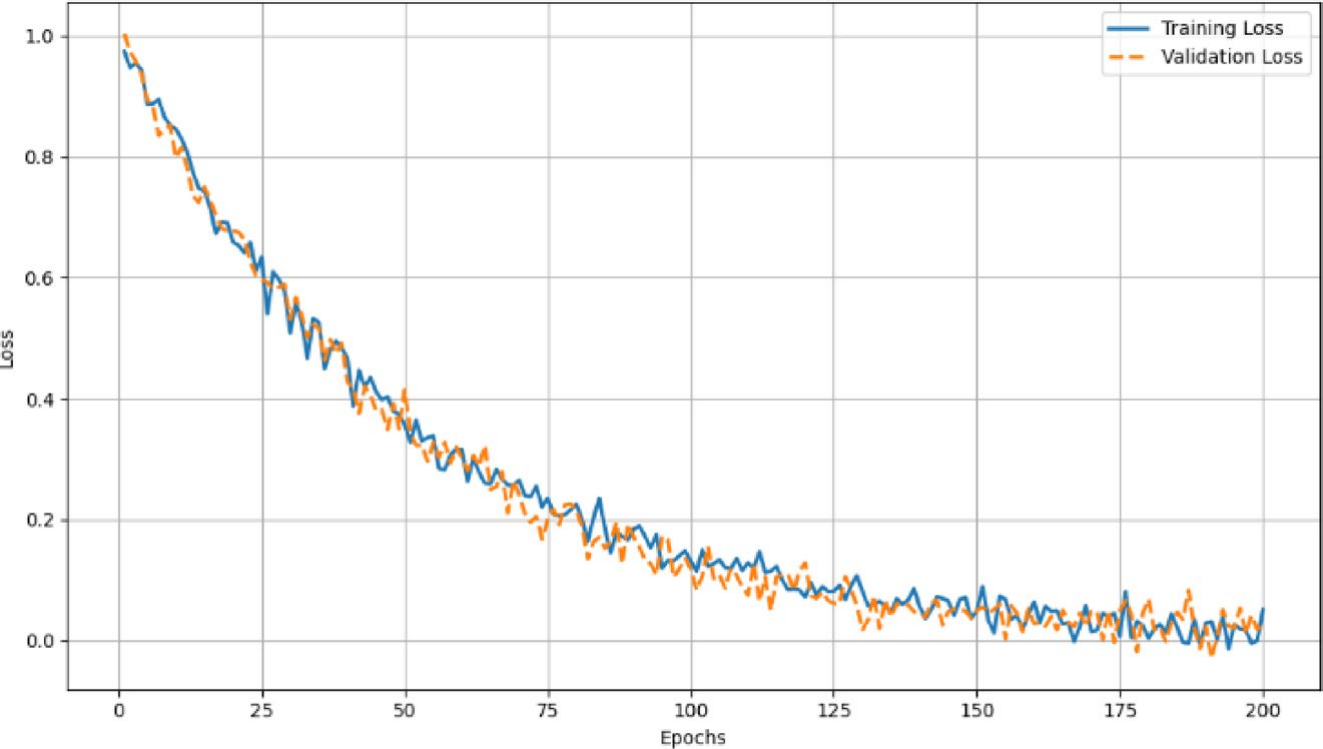
Loss curve for different epochs.

Average Precision values for all classes are listed in Table 4. The trade-off between Precision and Recall measures for all the classes are shown as PR curve in Fig. 11. The training loss and validation loss values for different epochs are measured and plotted in the Loss curve as Fig. 12.

**Table 4:** Average precision (AP) scores.

Class	Average precision (AP)
2–3 Wheelers	0.1220
Cars	0.1164
Buses	0.1464
Hatchbacks	0.1118
Sedans	0.1173
SUVs	0.0481
Tractors	0.1089
Light CVs	0.2754
Heavy CVs	0.1458

## Conclusion & future scope

The novel hybrid model containing Tiny YOLO and YOLOR was constructed in the edge layer. The traffic surveillance system with the hybrid vehicle detection and classification model analysed the traffic video captured from the cameras. The analyses produced the class and count of each vehicle on the road. The use of Tiny YOLO had faster frame processing rates and classified objects at faster rates. The accuracy of the results was further enhanced by the YOLOR. This hybrid model with very little latency is more suitable for real-time monitoring of road traffic. The analysed traffic volume data are sent to the cloud traffic control system. The use of F-RNN to fasten the decision-making and the traffic control outputs are sent to the designated signals. The proposed framework was tested on the dataset videos and found to be very effective in controlling the dynamics of road traffic. However, the video analysis was not tested for the poor-quality videos obtained in deterring environments like dark nights, rain, snow and so on. The proposed model lacks the capability of performing well in such deterred scenarios. Many external elements, including weather and dynamic diversions in the route, have an impact on traffic flow. The future research can be directed towards improvising the detection and classification capability from videos deterred due to rain, night and so on. Also, the model can be trained and tested on multiple datasets with videos captured at different angles and containing different kinds of vehicles with active traffic controlling features. GAN-based data augmentation techniques will be explored to address class imbalance and improve the robustness of the model across underrepresented categories.

## Data Availability

The datasets used and/or analysed during the current study available from the corresponding author on https://github.com/ksmalathy/Ensemble.git.
